# Pentose Phosphate Pathway Is Critical for Providing Energy by Bypassing 6-Phosphofructo-1-Kinase (PFK1) During Increased Neuronal Activity

**DOI:** 10.3390/metabo16050321

**Published:** 2026-05-12

**Authors:** Tibor Kristian, Jaylyn Waddell, Mary C. McKenna

**Affiliations:** 1 Department of Anesthesiology and the Center for Shock, Trauma, and Anesthesiology Research (STAR), University of Maryland School of Medicine, Baltimore, MD 21201, USA; 2 Department of Emergency Medicine, University of Maryland School of Medicine, Baltimore, MD 21201, USA; jwaddell@som.umaryland.edu; 3 Department of Pediatrics and Program in Neuroscience, University of Maryland School of Medicine, Baltimore, MD 21201, USA; mmckenna@som.umaryland.edu

**Keywords:** neurons, astrocytes, glycolysis, pyruvate, glucose metabolism, pentose-phosphate pathway

## Abstract

Glycolysis and the pentose phosphate pathway (PPP) are two metabolic pathways that play crucial roles in brain energy metabolism. The glycolytic pathway is differentially regulated in neurons compared to astrocytes. In neurons, the flux directly through the glycolytic pathway is reduced due to compromised ability to activate the key glycolytic enzyme 6-phosphofructo-1-kinase (PFK1). Consequently, potential increases in neuronal glucose metabolic flux can occur through the PPP, leading to the generation of NADPH, which is essential for the antioxidant defense system in these cells. Additionally, the PPP can supply glycolysis with intermediates downstream of PFK1, resulting in the production of pyruvate, which is used by mitochondria for oxidative phosphorylation and ATP production. In this review, we propose that during increased activity, neurons will preferentially metabolize glucose through the PPP. This allows them to support their antioxidant defense mechanisms and maintain bioenergetic metabolism by bypassing the limiting PFK1 enzyme and still forming pyruvate for mitochondrial oxidation.

## 1. Introduction

The brain is one of the most complex and energy-intensive organs, characterized by its integrated structure of neurons and glial cells, each with distinct energy demands. Consequently, the brain’s energy metabolism is unique, employing multiple metabolic pathways, and its function relies on a continuous supply of energy substrates, mainly glucose, but also ketones, fatty acids, glutamine, and lactate [1,2]. This is due to the limited internal energy stores of brain cells and the significant variation in energy requirement caused by neuronal activity. The majority of the energy utilized by the brain is derived from glucose metabolism via glycolysis and oxidative phosphorylation [2,3]. Glucose also serves as a substrate for the pentose phosphate pathway (PPP), which is a fundamental component of cellular metabolism, since it provides precursors for nucleotide and amino acid biosynthesis [4,5]. Additionally, the PPP is essential for maintaining cellular redox state by generating NADPH, which supports reduced glutathione and helps suppress oxidative stress [4].

Though both neurons and glia preferentially use glucose for energy production, during conditions of increased activity, energy production in different types of brain cells is dependent on specific metabolic pathways and/or substrates. This led to consideration of the intercellular shuttle of energy substrates between neurons and astrocytes, particularly glutamine [6] and lactate [7]. Although the notion of astrocyte-generated lactate driving neuronal activity is feasible, data directly supporting this idea are currently lacking [8]. Here, we propose an alternative hypothesis: increased glucose metabolic flux via PPP in neurons to support their energy demand during high activity.

While most cell types can use several substrates for energy production, the utilization of specific substrates and the maximal rate of oxidative phosphorylation are cell-type-dependent and determined by differential expression of transporters, metabolic enzymes, and respiratory complexes involved in related bioenergetic processes [2,9].

## 2. Glucose-Derived Brain Energy Metabolism

**Figure 1:**
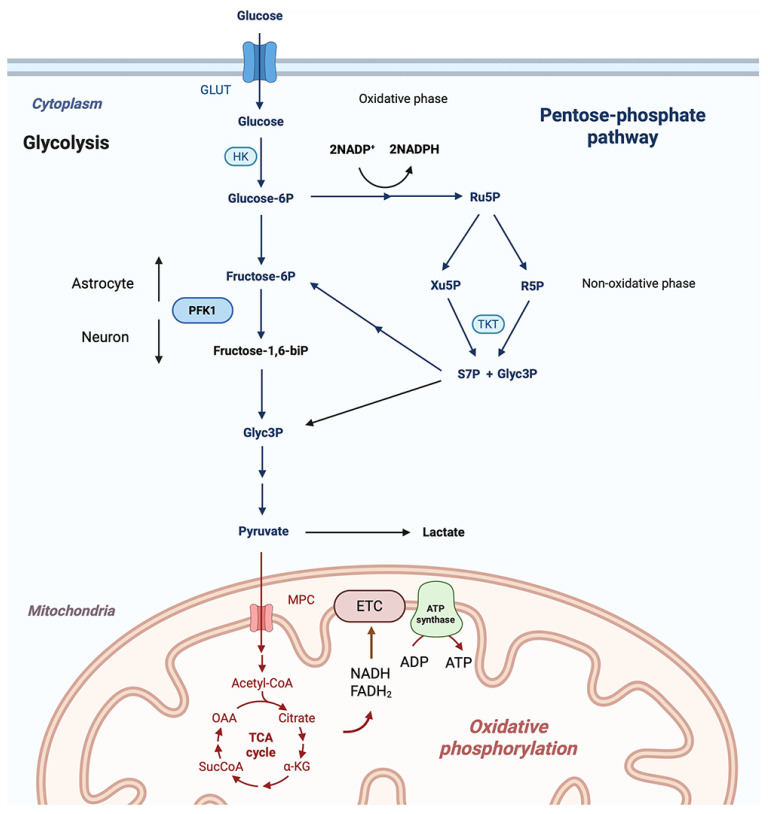
Glycolysis and the pentose phosphate pathway (PPP). Unlike in astrocytes, in neurons, the glycolytic flux rate modulation is limited. This is due to the limited generation of the stimulator that activates the glycolytic enzyme PFK1. Therefore, a potential increase in neuronal glucose metabolic flux can take place via the PPP. Following phosphorylation of glucose by hexokinase (HK), the glucose-6-phosphate either continues to be metabolized via the glycolytic pathway or enters the oxidative phase of the PPP that generates NADPH and ribulose-5-phosphate (Ru5P). The non-oxidative phase of the PPP can bypass the limiting step at PFK1 and supply glycolysis with intermediates derived from R5P, including Glyc3P, which is further metabolized to pyruvate and supports mitochondrial oxidative phosphorylation.

Glycolytic enzymes are localized in the cytosol, where glucose is metabolized to pyruvate [2]. In the first step, glucose is phosphorylated by hexokinase (HK), generating glucose-6-phosphate. Hexokinase 1 (HK1), one of the distinct isoforms of HK expressed in neurons, is predominantly localized to mitochondria [10,11]. This subcellular proximity to mitochondria suggests that HK1 activity is directly associated with mitochondrial oxidative phosphorylation [12] and also plays a role in modulating mitochondrial free radical generation [13,14,15]. The product of glycolysis, pyruvate, can be used by mitochondria for oxidative phosphorylation or converted to lactate (Figure 1). In mitochondria, oxidative phosphorylation is initiated by the transport of pyruvate into the mitochondrial matrix and its conversion to acetyl-CoA by the pyruvate dehydrogenase complex (PDH). During oxidation of pyruvate to acetyl-CoA, carbon dioxide (CO_2_) is released [16]. The acetyl-CoA enters the TCA cycle where in the first step it is condensed with oxaloacetate to form citrate (Figure 1). The TCA cycle produces reduced cofactors NADH and FADH_2_, which deliver their reducing equivalents to the electron transport chain (ETC) that uses oxygen as the final electron acceptor (Figure 1). During the flow of electrons via respiratory chain complexes, a mitochondrial membrane potential is generated that drives ATP synthesis. The net energy produced by the glycolytic pathway is only 2 ATPs per glucose molecule, compared to the 32 ATPs generated by oxidative phosphorylation in mitochondria [2]. In the brain, ATP is used to support complex neurological functions, including neuronal signaling, which consumes about 80% of the brain’s energy, and non-signaling activities that use 20% of the ATP generated by glucose metabolism [2,17].

The brain requires a continuous supply of oxygen and glucose due to high ATP demand. Thus, although the brain accounts for only 2% of body mass, it consumes 20% of total energy [17,18].

### 2.1. Cell-Type Specificity of Glycolytic Metabolism

Brain tissue is composed of two main cell types: neurons and glia. The most energy-intensive process in the brain is maintaining and restoring the neuronal membrane potential after increased neuronal activity [17].

Consequently, about 80% of the energy generated in brain tissue is utilized by neuronal cells [17]. The relatively low energy demand of astrocytes enables them to generate energy mainly by their glycolytic activity and oxidation of glutamate taken up from the synaptic cleft [19,20,21,22]. Thus, about 30% of oxidative metabolism in the brain in vivo occurs in astrocytes [23].

Interestingly, despite the high energy demand of neurons, these cells have a limited capacity to modulate glycolysis due to posttranslational downregulation of the glycolysis-promoting enzyme 6-phosphofructo-2-kinase-3 (PFKFB3) [24,25,26]. This enzyme generates fructose-2,6-bisphosphate, the main regulator of glycolysis, which activates the glycolytic enzyme 6-phosphofructo-1-kinase (PFK1) [27]. Neurons have lower levels of PFKFB3 compared to astrocytes due to its rapid neuronal degradation after ubiquitination by the anaphase-promoting complex/cyclosome (APC/C) [24,25], when bound to its activator CDH1 [28]. These in vitro findings were also supported by in vivo studies in a transgenic mouse model in which PFKFB3 was overexpressed in neurons [29]. Neurons in these mice showed bioenergetic deficiency, abnormal mitochondria, reduced NAD^+^ levels, and impaired autophagy [29].

Fructose-2,6-bisphosphate is one of many allosteric effectors that regulate PFK1, including citrate, AMP, ADP, ATP, and inorganic phosphate [30,31,32,33]. Recently, it was demonstrated that free cytosolic citrate in neurons declines rapidly several-fold upon neuronal activation and then gradually returns to baseline within several minutes, suggesting activation of TCA cycle enzymes and glycolysis [34]. However, the relative contribution of rapid decreases in citrate to activation of PFK1 will depend on the levels of other allosteric regulators, particularly the above-mentioned potent activator fructose-2,6-bisphosphate.

This raises the key question: “What do neuronal mitochondria use as a substrate during increased neuronal activity when the energy demand is significantly elevated?”

An astrocyte-to-neuron lactate shuttle (ANLS) mechanism was proposed, in which neuronal activity increases astrocytic glycolysis to support energy requirements for extracellular glutamate uptake, generating surplus lactate that is released by astrocytes and then taken up by neurons as an energy source [7,35,36]. This hypothesis, based primarily on in vitro studies, became one of the most controversial topics in brain energy metabolism. A more detailed discussion of controversies surrounding the ANLS can be found in several extensive reviews [8,37,38,39,40,41,42,43,44,45,46,47,48,49,50]. It is important to note that when multiple substrates are present in freshly isolated synaptic terminals from immature or adult rat brain, they preferentially use 3-hydroxybutyrate and glutamine rather than lactate as substrates [51].

### 2.2. Glucose Metabolism Through the Pentose Phosphate Pathway Can Support Neuronal Activity

Increased neuronal activity is associated with glutamate release and triggers elevated oxidative stress that can cause neuronal damage [52,53,54]. Neuronal cells are protected from free radicals by antioxidant mechanisms comprising enzymes that detoxify reactive oxygen species using reduced glutathione and NADPH [55,56,57]. To maintain the glutathione in its reduced form, neurons generate NADPH via glucose metabolism in the pentose phosphate pathway (PPP) [24].

The PPP branches from glycolysis at the first step, catalyzed by hexokinase, and uses glucose-6-phosphate (G6P) as its primary substrate (Figure 1). The PPP comprises two phases/branches: the oxidative and non-oxidative phases. The oxidative phase, which generates NADPH and ribonucleotides, has three reactions. The first and second reaction forms NADPH and the third forms ribulose-5-phosphate (Ru5P), which is then converted to ribose-5-phosphate (R5P) or Xylulose-5-phosphate (Xu5P) (Figure 1). The non-oxidative phase comprises a series of reactions that generate glycolytic intermediates, such as fructose-6-phosphate (F6P) via enzymes transketolase (TKT) and transaldolase. The transketolase enzyme activity also generates glyceraldehyde-3-phosphate (Glyc3P), another glycolytic intermediate [58,59].

Thus, this part of the PPP can lead to the production of pyruvate since the intermediate metabolite Glyc3P is further metabolized to pyruvate. However, importantly, it bypasses the glycolytic enzyme PFK1 (see Figure 1). The significance of this pathway activity for protecting neurons was confirmed by showing that redirecting most of neuronal glucose metabolism via the glycolytic pathway by overexpressing PFKFB3 led to increased oxidative stress and mitochondrial damage in cortical neurons [29].

### 2.3. Role of Glucose Metabolism via PPP in Astrocytes

Interestingly, although the PPP activity is essential for neurons, it has been shown that, in astrocytes, the PPP is also stimulated, leading to NADPH generation [60,61]. Under basal conditions in vitro, glucose flux into the astrocytic PPP was five to seven times higher than that into the neuronal PPP [62,63]. Astrocytes play important roles in defense against oxidative stress [64,65], as reflected in their high glutathione (GSH) levels [66,67] and in their ability to supplement neurons with precursors for GSH synthesis [68,69,70]. Furthermore, higher NADPH levels in astrocytes support lipid and cholesterol syntheses [71]. After glutamate exposure of astrocytes, both glucose utilization and PPP flux significantly increase, indicating that neuronal excitation activates PPP in both astrocytes and neurons [72]. Thus, since astrocytes show high levels of glutathione and their PPP activity is several-fold higher than that of neurons under basal conditions [62,73], during high energy demand, the glycolytic glucose metabolic flux can increase in astrocytes without compromising the cells’ antioxidant protection.

### 2.4. Regulation of PPP

In the brain, PPP generally contributes a minor fraction of the total intracellular pyruvate generated compared to glycolysis, typically ranging from 3% to 6% of glucose metabolism in resting brain tissues [74]. In neurons, the basal PPP contribution is very low, estimated at roughly 0.25% to 3.5% of total glucose metabolism [75]. In glial cells, this can be higher, around 6.9%. However, under conditions of high oxidative stress, cells can divert up to 22–66% of glucose through the PPP to maximize NADPH production [60,75,76].

Thus, the activity of the PPP is rapidly augmented when cells are exposed to free radicals or when reactive oxygen species (ROS) production is increased. This response also involves metabolic and gene regulatory mechanisms [4]. Immediately after the increase in oxidative stress, enzymes of glycolysis, glyceraldehyde 3-phosphate dehydrogenase (GAPDH) and pyruvate kinase (PK) are inhibited [77,78], while the flux via PPP continues [78,79,80]. This is followed by a transcriptional response that leads to up-regulation of PPP enzymes and their posttranslational modifications [80,81]. Thus, transcriptional regulation of G6PDH is induced by oxidative stress and by the need for NADPH and PPP intermediates for anabolic reactions, such as lipid and nucleotide synthesis [82,83]. Regulation during oxidative stress is mediated by the Kelch-like enoyl-CoA hydratase-associated protein 1 (Keap1)/nuclear respiratory factor 2 (Nrf2) system [84,85]. Nrf2 controls the transcription of a wide spectrum of antioxidant enzymes, including those required for the GSH pathway and for NADPH regeneration [86,87]. Under normal resting conditions, Nrf2 is anchored with its adaptor protein Keap1. The Keap1/Nrf2 complex prevents Nrf2 from being translocated into the nucleus, thereby inhibiting its transcriptional activity. Reactive oxygen species react with the thiol of the cysteine residue of Keap1 and induce the release of Nrf2 from Keap1. Free Nrf2 molecules translocate into the nucleus and initiate the transcription of target genes.

Generally, under physiological conditions, the PPP has lower activity than glycolysis, accounting for only 2–5% of total glucose consumption [88,89,90]. However, the PPP enzymes have a large excess capacity, and flux through this pathway has been shown to increase robustly during oxidative stress [91,92].

**Figure 2:**
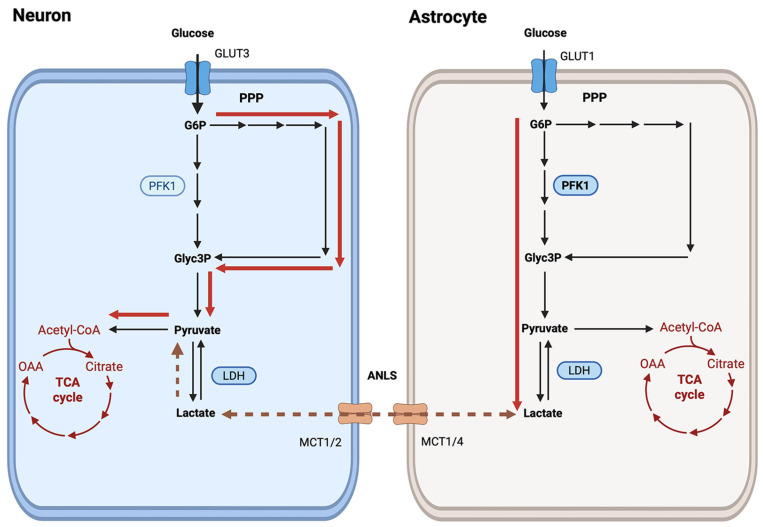
Schematic diagram of glucose metabolic flow in neurons and astrocytes during increased neuronal activity. After glucose is transported into neurons and astrocytes by glucose transporters (GLUT3) and (GLUT1), glycolysis produces pyruvate, which supports mitochondrial energy production and the tricarboxylic acid (TCA) cycle metabolism. In neurons, high neuronal firing leads to oxidative stress associated with a reduction in the NADPH/NADP ratio and stimulation of the pentose phosphate pathway (PPP). Glyceraldehyde-3-phosphate (Glyc3P) generated by the PPP can be further metabolized to pyruvate to support ATP production by neuronal mitochondria. Bolded lines represent the metabolic flux during increased neuronal activity. During enhanced glucose metabolism in astrocytes, the increased glycolytic rate results in excess lactate production. Lactate diffuses along the concentration gradient into the intracellular fluids via monocarboxylate transporters 1 and 4 (MCT1/4) and can be removed from brain tissue via blood or taken up into neurons through MCT1/2. Lactate dehydrogenase (LDH) can oxidize lactate to pyruvate. The dashed line represents the lactate movement between neurons and astrocytes.

Thus, the pentose phosphate pathway has excess capacity to increase its flux [93], particularly in neurons, since baseline activity is very low in these cells [62,63]. Metabolic flow through the PPP is specifically modulated by glucose 6-phosphate dehydrogenase (G6PD), which depends on the NADPH/NADP ratio [4]. Therefore, increased oxidative stress leads to reduced NADPH levels, and the glucose metabolic flow via the PPP pathway is stimulated [5]. This suggests that during high activity, neurons use glucose as an energy substrate, metabolizing it via the PPP (Figure 2). Although lactate can also serve as an energy substrate for both neurons and glia, the specific cell type that will utilize lactate depends on its intra- and extracellular concentration gradient and on the cytosolic NADH/NAD ratio that will affect the lactate oxidation rate by lactate dehydrogenase (LDH) [40]. Lactate can indeed support energy metabolism, and either cell type may consume it when available [1,21], or it can leave the brain via the blood [19,94]. Release of some lactate from the brain is necessary to balance the carbons added via anaplerosis [95]. Conversion of lactate to pyruvate consumes energy and requires a functioning malate-aspartate shuttle in neurons [96]. However, to protect the neuron during increased activity, the metabolic flux of glucose via PPP maintains the delivery of pyruvate to PDH for subsequent oxidative phosphorylation and supports cellular antioxidant activity. This is consistent with reports of increased glucose metabolism in neurons upon stimulation [50,97,98,99,100,101]. Interestingly, energy demand during long-term memory formation leads to glucose shuttling from glia to neurons to fuel the neuronal PPP [102]. Thus, prolonged neuronal glucose consumption has a critical role during long-term memory formation and is supported by a glucose shuttle between glial cells and neuronal somata during the early phase of memory consolidation [102].

### 2.5. Brain Region-Dependent PPP Activity

Pentose phosphate pathway activity is not uniform across different brain regions and cell types [4]. The differences in PPP activity between individual brain structures are driven by regional differences in metabolism, cell type composition, and functional demand [103]. Since glucose metabolism is region-specific, the PPP is highly active in the thalamus [103], while glycolysis tends to predominate in the neocortex [103]. Within the neocortex, a high metabolic profile is observed, with glucose metabolism primarily focused on supporting high synaptic activity and redox defense, particularly in neurons [104]. Some cortical and limbic regions show relatively high PPP gene expression, as shown by recent whole-brain transcriptomic maps that report distinct regional distributions of PPP versus other energy pathways (glycolysis, TCA cycle, and lactate metabolism) [104].

Similarly, the hippocampus, critical for memory, relies on PPP activity for long-term memory formation [105]. Studies on hippocampal slices show that inhibiting the PPP increases reactive oxygen species (ROS), highlighting its role in antioxidant defense, which may vary depending on neural activity in different hippocampal subfields [106].

### 2.6. PPP Activity During Disease Conditions

Under pathologic conditions or after brain insults, PPP flux increases, particularly when oxidative stress is induced [107,108]. For example, brain ischemia generally upregulates the PPP as a protective mechanism against oxidative stress. By increasing the activity of the rate-limiting enzyme G6PD, the brain boosts production of NADPH, which helps regenerate reduced glutathione to combat free radicals and reduce neuronal death [107,109,110].

Since hyperglycemia is associated with increased oxidative stress, high glucose levels also activate the PPP [62]. Furthermore, PPP activity is vital for peripheral nerve regeneration. The PPP is enriched and active in sciatic nerve axoplasm to maintain NADPH levels [111]. Injury to the sciatic nerve leads to inhibition of PPP, consequently reducing ribonucleotide production via ribose-5-phosphate. Reactivation of the PPP by overexpression of transketolase or oral ribose supplementation promotes sciatic nerve recovery [111].

Glucose can be transported from blood or generated in cells by glycogen breakdown, particularly during periods of food deprivation or stress [112]. Glycogen phosphorylase is the rate-limiting enzyme in glycogen metabolism [113].

Interestingly, there is a distinct signature of impaired glycogen metabolism in the brain of Alzheimer’s disease patients, indicating a link between tauopathies and glycogen metabolism. Breakdown of neuronal glycogen by activating glycogen phosphorylase redirects glucose flux to the PPP, alleviating oxidative stress and ameliorating tauopathy symptoms [114]. Furthermore, disruption of glycogen metabolism also occurs in epilepsy and type 2 diabetes [115].

## 3. Conclusions

Brain glucose metabolism exhibits considerable variability, influenced by factors such as brain region, cell type, and neuronal activity or stress. The complex interactions between neuronal and glial cells, along with the specialized functions of different brain cell types, require the use of specific metabolic pathways and substrates, especially during periods of increased energy demand. Although various metabolites are exchanged between neuronal and non-neuronal cells, the concentration gradients of lactate within and between cells play an important role in regulating the direction of its exchange. The transport rate is also modulated by the expression levels of the corresponding transporters [116,117].

The differential expression of glycolytic and oxidative phosphorylation (OXPHOS) enzymes between neurons and astrocytes likely evolved to facilitate glucose metabolism through the PPP. This pathway enables neurons to bypass the limitation in glycolytic flux modulation at PFK1 and provides antioxidant protection, thereby safeguarding neuronal cells during periods of high energy demand. Consequently, the PPP-linked increased pyruvate generation can also contribute to energy supply for neurons with high activity.

## Data Availability

No new data were created or analyzed in this study.

## Abbreviations

The following abbreviations are used in this manuscript:

ANLS	Astrocyte-to-neuron lactate shuttle
APC/C	Anaphase-promoting complex/cyclosome
ATP	Adenosine triphosphate
ETC	Electron transport chain
F6P	Fructose-6-phosphate
G6P	Glucose-6-phosphate
G6PD	Glucose-6-phosphate dehydrogenase
GAPDH	Glyceraldehyde 3-phosphate dehydrogenase
GLUT	Glucose transporter
Glyc3P	Glyceraldehyde-3-phosphate
GSH	Glutathione
HK	Hexokinase
Keap1	Kelch-like enoyl-CoA hydratase-associated protein 1
LDH	Lactate dehydrogenase
MCT	Monocarboxylate transporter
MPC	Mitochondrial pyruvate carrier
NADPH	Nicotinamide dinucleotide phosphate
Nrf2	Nuclear respiratory factor 2
OAA	Oxaloacetic acid
PDH	Pyruvate dehydrogenase complex
PFK1	6-phosphofructo-1-kinase
PFKFB3	6-phosphofructo-2-kinase-3
PK	Pyruvate kinase
PPP	Pentose-phosphate pathway
ROS	Reactive oxygen species
Ru5P	Ribulose-5-phosphate
R5P	Ribose-5-phosphate
S7P	Sedoheptulose 7-phosphate
TCA	Tricarboxylic acid cycle
TKT	Transketolase
Xu5P	Xylulose-5-phosphate
α-KG	α-ketoglutarate
